# Scarlatina Treatment and Results

**Published:** 1889-11-09

**Authors:** 


### SCARLATINA TREATMENT AND RESULTS.

It has fallen to the lot of few men to have treated in hospital and in private practice 3,640 cases of scarlatina, which is the record in 20 years of Dr. Reimer, house physician at the Nikolaëvsky Hospital for Sick Children at Petersburg. In a paper read at the congress of Russian medical men held last summer, he gave analyses of 2,868 cases arranged under eight groups, based on the duration and severity of the disease, and the presence or absence of complications; with the ratio each bore to the total and the mortality in the several groups. Thus 61, = 1·1 per cent., were mild and uncomplicated, with a mortality of 0. Severe and uncomplicated, 211, = 5·7 per cent., mortality 83·7 per cent. The cases attended with complications were 2,596. Of these there were of mild character and moderate duration 317, = 9·1 per cent., and a mortality of 2 per cent. Graver, but of moderate duration, 468, = 13·5 per cent., mortality 7 per cent. Mild, but rather protracted, 339, = 9·7 per cent., mortality 5·6 per cent. Severe, and rather protracted, 682, = 18·7 per cent., mortality 39·3 per cent. Mild, but prolonged, 419, = 12·1 per cent., mortality 26·5 per cent. Severe and prolonged, 371, = 10·7 per cent., mortality 80 per cent. He next discussed the loss of weight, based on 18,976 weighings of 968 cases, of which 344 were treated in the expectant plan, and 624 by antipyretic measures. In mild cases without treatment the loss is trifling; in severe ones it may reach 600 to 1,000 grams during the efflorescence, and 2,000 to 2,500 or more in seven to ten days; while in protracted cases the child may lose in a minute as much as one-seventh of its original weight. In those marked by a constant high temperature drugs proved powerless to check the loss of weight, but some controlling influence appeared to be exerted by hydrotherapeutic measures.

The most valuable portion of his report deals with the effects of the several hydrotherapeutic methods and antipyretic drugs on the temperature, nervous symptoms and the action of the heart, containing much of suggestion as well as of caution in these days of heroic antipyresis, his conclusions being drawn from data numerous enough to eliminate the too common "error of insufficient observation." The cases were all of a grave and hyperpyretic character, 978 being treated by hydrotherapeutic measures and 1,869 with antipyretic drugs. These together amount to 2,847 cases, the 793 unaccounted for including those treated expectantly or before antipyretic measures or drugs had come in vogue. Of the first group of 978 cases, 102 were treated by cold compresses to the head, chest, or abdomen, as well as cold ablutions of the whole body. These were found to exert a sedative action on the nervous system, but to have no influence on the course of the fever itself. Cold packs were tried in 28 cases, with intense nervous excitement. They failed altogether to reduce the temperature, and were occasionally followed by a further rise, not unfrequently by cyanosis and failure of the pulse, and if prolonged for several hours even by fatal collapse. Cold packs, with cold irrigation, 131 cases, were more satisfactory in every respect, but showed a very trifling influence on the fever. The same might be said of the ninety-six cases in which cold packs with cold irrigation were employed in a gradually cooled bath; they, however, acted more rapidly, but too frequently were followed by collapse. Tepid baths, seventy-two cases, were useless, and if prolonged for half an hour or more, dangerous, inducing weakness of the pulse and cyanosis, which could, however, be obviated by cold irrigations. Baths, at 95 F., gradually cooled down to 80 F., in 186 cases proved more frequently fatal from collapse and sudden failure of the heart than any other application of the kind, whereas by far the most satisfactory results were obtained by cold baths, at 64deg., 68deg., or 72deg. F., of five to eight minutes' duration, accompanied with vigorous friction of the whole body. This treatment was employed in 363 cases, effecting a reduction of temperature of 3deg. to 4deg. F., with considerable improvement of the pulse and respiration.

These latter observations are of special interest to the general practitioner who has to take account of the prejudices and apprehensions of the parents, since he may confidently assure them, as we have often done, that the cold bath, though apparently the most heroic, is the safest, being, in fact, devoid of danger and productive of positive benefit; whereas what would seem to be the gentler means are, for the most part, useless or dangerously depressing. It is, in fact, the stimulating action of the cold bath that constitutes its superiority.

The results obtained by Dr. Reimer from drugs were less definite, and not such as to encourage one in employing them; although patients and their friends have no objection to "putting drugs of which they know little into bodies of which they know less," and, we may add, demur only to those drugs whose physiological action is most evident on the assumption that such manifest effects involve a degree of danger. Dr. Reimer tried quinine in 148 cases, but whether he administered it by the mouth, or in enemata or hypodermically, could not detect any influence on the temperature during the period of efflorescence, and but little during defervescence. Salicylate of soda, given in 431 cases, was absolutely inert as regards the temperature, but decidedly injurious in its action on the heart. Kairin, tried in 36 cases, was worse, depressing the respiratory functions also, while exerting scarcely any influence on the temperature. Thallin, in 48 cases, was uncertain in the extreme, sometimes having no action whatever on the temperature, and at other times or in other cases causing an excessive fall and alarming collapse. Antipyrin, a far safer drug, was given in as many as 684—or more than one-third of the cases. It exhibited no effect on the temperature during efflorescence, nor did it in any way modify the course of the disease. Still, to quote Dr. Reimer's own words, "it proves to be distinctly beneficial in protracted cases, in virtue of its successfully counteracting the injurious effects of tissue combustion." He probably means the toxic action of the products of albumenous metabolism on the nervous centres, and "it seems to enable children to cope better with severe complications of scarlet fever." Altogether it is preferable to "other antipyretic drugs, its main advantage being a comparative freedom from unpleasant accessory effects." Antifebrin, used in 522 cases, resembled antipyrin in its action, but required greater caution, more frequently producing unpleasant symptoms, especially cyanosis and collapse in patients suffering from cardiac affections.

The general conclusion to be drawn from this enormous mass of observation and experiment appears to us to be that: 1. The best, as being at once the most certain and the safest means of reducing hyperpyrexia, is the cold bath (60deg., 65deg., 70deg.), maintained for about five minutes, with friction, if the heart be weak, though cold sponging may be at least grateful in milder cases. 2. That cold, in the form of the ice bag to the head, may be employed to quell delirium. 3. That no drug can be relied on as an antipyretic, but that antipyrin is a valuable sedative to nervous excitement, and 4. That it, and quinine, may be usefully combined after the subsidence of the eruption. 5. That it is important to employ cardiac stimulants, such as digitalis or strophanthus, with ether or alcohol, in threatened failure of the heart; ammonia being, we believe, undesirable. 6. Local antiseptic applications by means of spraying to the throat and fauces may obviate putrid absorption; for this purpose we ourselves prefer the non-irritating liq. pot. permang. to stronger chemicals, since a local germicide is not indicated, as in diphtheria, and sucking ice is a useful and agreeable adjunct. 7. We may also add that in hyperpyrexia and intense cerebral excitement we have found affusion of the head with water not above 60deg. during the cold bath brilliantly successful. 8. Lastly, that so long as hyperpyrexia persists, no fears need be entertained of renal mischief being induced by the use of cold in the ways above recommended.

